# Retrotransposon-Encoded Reverse Transcriptase in the Genesis, Progression and Cellular Plasticity of Human Cancer

**DOI:** 10.3390/cancers3011141

**Published:** 2011-03-07

**Authors:** Paola Sinibaldi-Vallebona, Claudia Matteucci, Corrado Spadafora

**Affiliations:** 1 Department of Experimental Medicine and Biochemical Sciences, University ‘Tor Vergata’, Rome, Italy; E-Mails: sinibaldi-vallebona@med.uniroma2.it (P.S.-V.); matteucci@med.uniroma2.it (C.M.); 2 Italian National Institute of Health (ISS), Rome, Italy

**Keywords:** tumorigenesis, endogenous reverse transcriptase, reverse transcriptase inhibitors, early embryonic development

## Abstract

LINE-1 (Long Interspersed Nuclear Elements) and HERVs (Human Endogenous Retroviruses) are two families of autonomously replicating retrotransposons that together account for about 28% of the human genome. Genes harbored within LINE-1 and HERV retrotransposons, particularly those encoding the reverse transcriptase (RT) enzyme, are generally expressed at low levels in differentiated cells, but their expression is upregulated in transformed cells and embryonic tissues. Here we discuss a recently discovered RT-dependent mechanism that operates in tumorigenesis and reversibly modulates phenotypic and functional variations associated with tumor progression. Downregulation of active LINE-1 elements drastically reduces the tumorigenic potential of cancer cells, paralleled by reduced proliferation and increased differentiation. Pharmacological RT inhibitors (e.g., nevirapine and efavirenz) exert similar effects on tumorigenic cell lines, both in culture and in animal models. The HERV-K family play a distinct complementary role in stress-dependent transition of melanoma cells from an adherent, non-aggressive, to a non-adherent, highly malignant, growth phenotype. In synthesis, the retrotransposon-encoded RT is increasingly emerging as a key regulator of tumor progression and a promising target in a novel anti-cancer therapy.

## 1. The Complexity of the Cancer Genomic Landscape

Historically, the concept that the genome plays a central role in cancer has been proposed since the early 20th century, when Boveri first identified chromosome alterations as characteristic features of dividing cancer cells. That view has been progressively strengthened with the discoveries that DNA represents the chemical basis for heredity [1], that DNA-damaging agents cause cancer [2] and that chromosome aberrations correlate with hematological malignancies [3]. Some time later, in an ideal continuity with those early findings, tumor suppressor genes [4] and oncogenes [5] were discovered, the transforming potential of which can be triggered by single base substitutions. The general view was established that cancer is the pathological consequence of the sequential accumulation of DNA mutations in relevant genes. Consistent with this view, a central objective in cancer research in the last decades has been the search for common recurrent mutations, shared by the genomes of a variety of tumors, that may provide key “signatures” of tumorigenic phenotypes [6].

Recent technological advances, such as next-generation genome sequencing [7] and DNA-based array analysis [8], have boosted that search, speeding up and empowering the ability to detect alterations (including copy number variation, indels of various sizes and single base substitutions) in the DNA of cancer cells. These efforts ultimately aimed to produce a catalog of the complete collection of “driver” somatic DNA mutations that could confer selective growth advantage and facilitate the clonal expansion of cancer cells in a Darwinian evolutionary view, as opposed to neutral “passenger” mutations accidentally caused by repair mechanisms [6,9]. The characterization of driver mutations and the identification of cancer-relevant genes targeted by these mutations were expected to be key to define the structure of the “cancer genome”, permissive for cancer onset, growth and propagation.

Despite the enormous amount of sequence information and microarray data accumulated in the last few years, however, these expectations have not been fulfilled. Shared or recurrent mutations that may potentially cause cancer have not been recognized as a predominant feature in large panels of tumors, but only in few specific cases [10–12]. In addition, the number of “cancer genes” is continuously growing in parallel with the expansion of the genome screening power [13]. In even sharper contrast with expectations, these efforts are unexpectedly revealing that genomes of any tumor type exhibit, in fact, remarkable heterogeneity with infrequent driver mutations or rearrangements [14,15].

The trend emerging from these studies is heavily influencing clinical oncology and the design of therapeutic approaches. There currently is an effort towards profiling individual cancer genomes, identifying new genes and accurately dissecting mutation-harboring networks within tumors at the individual patient level in an attempt to define promising grounds for the design and improved outcome of therapy [16]. These findings, on the one hand, contribute to expand our knowledge of cancer genetics; on the other hand, they raise more questions than they can answer. A major unanswered question is whether a unifying meaning can be derived from such a flood of sequence data, given the failure to identify “universal” driver mutations or key targeted genes in the onset and progression of cancer. Another puzzling question is how the recurrent biological and clinical patterns characterizing most, if not all, cancers (uncontrolled proliferation, loss of differentiation, loss of contact inhibition, invasiveness) can be reconciled with the genetic heterogeneity of potential causative mutational events.

It is important to note that large-scale genomic studies have focussed on protein-coding genes, which account for a mere 1.2% of the human genome [17]. The non-coding portion, which constitutes the vast majority of the genome and presumably harbors a higher amount of mutations, has tended to be excluded. That portion of the genome has long been dismissed as selfish [18,19] or ‘junk’ DNA. It has only being “rehabilitated” in recent studies that have disclosed global regulatory roles for small RNA families [20], non-coding RNA (ncRNAs) [21,22], ultra conserved regions (UCRs) [23,24] and retroelements [25,26]. These studies are revolutionizing current views on genome function and are disclosing unexpected implications for tumorigenesis [27–30].

In this framework, our own studies in the last few years have pinpointed a role played by an endogenous reverse transcriptase (RT) encoded by LINE-1 (Long Interspersed Nuclear Elements) retrotransposons in loss of differentiation of cancer cells and tumor progression. That work stemmed from earlier findings that spermatozoa and early embryos are endowed with an endogenous RT activity that is essential for embryo development. What follows is an account of research on the genome-shaping roles of RT from gametes through embryos to tumor cells.

## 2. Retrotransposon-Encoded RT in Spermatozoa, Early Embryos and Transformed Cells

RT-encoding genes are abundantly represented in eukaryotic genomes and are harbored within two large families of autonomously replicating retroelements, LINE-1 and HERV (Human Endogenous Retroviruses). Together with the non-autonomous Alu and SINE retrotransposon families, retroelements account for an amazing 45% of the human genome [17,31] and constitute the bulk of the genome portion that was historically called “junk DNA” [18,19]. The RT enzyme operates a fundamental step in the amplification of retrotransposons and thus constitutes the main driving force for their accumulation throughout evolution. Due to this predominant role, RT has not been traditionally thought of as a candidate for any other physiological function of any potential interest to cell life.

Our unexpected finding that mouse spermatozoa are endowed with an endogenous RT activity [32] changed that view and provided the first hint that this enzyme may play other roles. Surprisingly, we found that RT localizes in mature sperm heads, where fertilizing functions reside. That finding raised the intriguing question of whether RT is involved in early embryonic development. Indeed, we found that mouse early embryos at the one-, two- and four-cell stages contain an endogenous RT activity able to reverse transcribe exogenous RNA molecules into cDNA copies [33]. Importantly, inhibition of that RT activity, induced either by a non-nucleoside RT inhibitor [33], (nevirapine, used in AIDS therapy, [34]), or by down-regulating RT-encoding gene expression via antisense oligonucleotide microinjection into zygotes [35], arrested development drastically and irreversibly at the two- and four-cell stages. Thus, the embryonic RT, far from being a useless evolutionary remnant encoded by “parasitic” genetic elements, is an essential player in preimplantation development. That work also identified the major source of functional embryonic RT activity in a specific LINE-1 retrotransposon family [35], previously characterized as highly active in mouse cells [36].

Several studies have expanded the notion that retrotransposition is an ongoing process in human cells, whose genome is continuously being reshaped [30,37,38]. Among hundred of thousands of LINE-1 elements present in the human genome, only a few are full-length and retrotranspositionally competent. These elements constitute an active subpopulation responsible for the vast majority of all LINE-1-mediated retrotransposition events [39]. LINE-1 retrotransposition has been shown to actively occur essentially in embryos [40], in good agreement with our own data.

It is a long-standing notion, dating back to the middle of 19th century, that embryogenesis and tumorigenesis share common functional features [41] and that the process of tumor growth reflects in some case the reactivation of embryonic programs [42]. In this light, it was relevant to establish whether tumor cells are endowed with RT activity, similarly to embryos. We therefore extended the search for this activity to a variety of murine and human tumorigenic cell lines using the same functional assay we had used with embryos. We found that, regardless of the type or histological origin of tumors, all analyzed cell lines contained appreciable levels of RT activity [43]. Importantly, preincubation of cell lysates with nevirapine drastically reduced their ability to reverse-transcribe RNA templates *in vitro*, suggesting that the RT of tumorigenic cells is sensitive to pharmacological RT inhibitors. The latter finding provided us with a tool to assess the role of the endogenous RT in the genesis and progression of human tumors.

## 3. Reverse Transcriptase Plays a Causative Role in Tumorigenesis

RT inhibitory drugs such as nevirapine and efavirenz (another nonnucleoside RT inhibitor employed in AIDS therapy [44]) induce three major effects in tumor cell lines and *ex vivo* patient-derived tumor cells: (i). reduced rate of proliferation; (ii). induction of cell differentiation; and (iii). global reprogramming of gene expression. These effects are induced rapidly (after only a short treatment with RT inhibitors for a few days) and are not restricted to specific cell lines, but are very widespread among transformed cell types [43].

In-depth studies of cell lines representative of different tumor types are summarized in Table 1: RT inhibition consistently triggered their differentiation, revealed by changes in cellular morphology and restored expression of typical differentiation markers of the healthy counterpart of the tumors.

Recent microarray-based analyses of A-375 melanoma cells before and after exposure to RT inhibitors suggest that reprogramming is not restricted to just a few markers but has a global impact, involving three large classes of nuclear transcripts: Protein-encoding mRNAs, micro RNA (miRNAs) and RNA transcribed from ultraconserved regions (UCRs). Importantly, all observed effects (reduced proliferation, morphological changes, induction of markers and gene expression reprogramming) are reversible on discontinuation of the RT-inhibitory treatment [45]: thus, the RT-dependent changes are not constitutively inherited through cell division, but are maintained as long as the RT is under continuous inhibition. This suggests that RT operates in an epigenetic nature.

These findings opened up the perspective of employing RT inhibition in anti-cancer therapy. To validate this idea, *in vivo* assays were carried out in murine models. Human tumorigenic cells (A-375 melanoma, PC3 prostate carcinoma, H69 small cell lung carcinoma and HT29 colon carcinoma) were inoculated in nude mice; one week later the RT-inhibitory treatment started. Consistent with the *in vitro* results, RT inhibition antagonized the progression of all four tumors [45]. Tumor progression was, however, quickly resumed on discontinuation of the treatment, confirming the epigenetic nature of the RT-dependent mechanism. An important conclusion emerging from that work is that prolonged RT inhibition does not eradicate the tumor, but rather maintains it in a repressed, non-invasive, state.

Conclusive evidence that tumors rely on high RT activity, and their treatment is truly due to RT inhibition, still required that non-specific off-target effects of efavirenz be ruled out. Work from Haig Kazazian's laboratory had identified and characterized a full-length, transcriptionally active and retrotransposition-competent LINE-1 family [39]; that family is regarded as the main source of RT activity and accounts for most of the retrotransposition activity occurring in human cells. Based on this identification, we performed RNA interference (RNAi) to down-regulate the expression of that particular LINE-1 family in A-375 cells. In a first set of experiments, we induced transient acute down-regulation of the highly active LINE-1 family by transfecting small interfering oligonucleotides (siRNA) [45]; in a second set, we infected A-375 cells with a retroviral vector stably expressing LINE-1-interfering construct [47]. Both approaches induced phenotypic changes in melanoma cells, *i.e.* reduced proliferation and differentiated morphology, consistent with those induced by pharmacological RT inhibitors. Furthermore, LINE-1-specific interference drastically reduced the tumorigenic potential of melanoma cells inoculated in nude mice [47]. These results confirm the essential role of LINE-1-encoded RT in tumorigenesis.

The correlation between retrotransposon activation and cancer is a long-standing notion, empirically established in several cancers (summarized in Table 2; also see [48–50]).

It was not clear, however, if the functional awakening of these elements is the consequence or the cause of cell transformation and tumor onset. The evidence summarized above suggest a causative role for LINE-1-encoded RT in tumor onset and progression, because LINE-1 inhibition is sufficient to revert transformed cells to a differentiated condition and to antagonize tumor growth. This conclusion fits well with earlier groundbreaking findings that have indicated a major role for genomic hypomethylation in tumor formation [57]. Alterations in DNA methylation patterns occur early in the process of transformation [58] and follow non-random, tumor-type–specific patterns [59]; they usually entail an overall decreased level of 5-methylcytosine contrasting with the hypermethylation of specific genes [50,60]. Relevant to the central argument of this review, DNA hypomethylation of cancer genomes largely affects repeated sequences and transposable elements; it is associated with heterochromatin decondensation and retrotransposon activation, including increased LINE-1 expression. This is seen as a route towards chromosomal instability and increased chromosomal recombination, and hence increased mutation rates. These studies, from a completely different angle, are therefore consistent with the view that retrotranposon activation is an early event in tumorigenesis.

How are the resumption of retroelement function and reactivation of RT-encoding gene expression initiated in somatic cells? Among possible initiators, cellular stress plays an important role. Retrotransposons are indeed DNA elements with a distinct sensitivity to environmental changes, which effectively promote their activation [61]. Growing evidence points to an ability of environmental stresses (e.g., oxidative and genotoxic stress, UV light, ionizing radiations, environmental hydrocarbons, infection and injury) to modulate non-coding RNAs, DNA methylation, histone modifications and chromatin remodeling, which ultimately determine the global cell fate and function [62,63]. Stress signals, therefore, can orchestrate global and mutually interplaying responses, entailing an overall hypomethylated genome status and histone acetylation at particular domains, which lead to the activation of retroelements [61,64,65].

Active retrotransposons contribute in turn to heterochromatin formation and modulate the overall organization of large chromosomal domains, thus modulating the expression of host genes at both the genetic and epigenetic level [25,26,66]. This role is well documented in studies of highly regulated processes during development. Transcriptional interference exemplifies a powerful epigenetic mechanism for RT-dependent control of gene expression [67]. Transcriptional interference indicates the interference exerted by active retroelements on neighboring genes in a mosaic pattern, depending on the expression level of the retroelement. The murine *agouti* gene is a typical case: expression of this locus is modulated by an LTR-containing a IAP (intracisternal A particle) retrotranposon element placed 100 kb upstream. When the IAP is silent, the *agouti* locus is expressed, but when IAP is active the expression of *agouti* is abrogated or proportionally down-modulated: thus, the IAP exerts a *cis*-acting control on a heterologous locus in a distance- and expression-dependent manner. Retrotransposon activity also modulates large-scale silencing processes, as in X chromosome inactivation in female cells [68]. In that case, a LINE-1-dependent mechanism directs the assembly and spreading of heterochromatin domains, with the ensuing creation of nuclear compartments into which genes are silenced. In cancer cells, therefore, interplay between retrotransposition and overall genome organization can be initiated in response to stress and determines changes in the global expression pattern and fate of the cells. In this light, retroelements can be viewed as “molecular rheostats” that can sense environmental stimuli and modulate gene expression profiles in response, thus determining the emergence of altered phenotypes and pathologies including cancer.

The very same ability of retrotransposons to be part of genome-reshaping mechanisms in response to external stimuli renders their mobilization a potential continuous threat to genome function. In evolutionary terms, cells have developed a variety of repressive mechanisms to restrict or abrogate their undesired mobilization and minimize their potentially adverse effects [69,70]. First, DNA hypermethylation was the first discovered epigenetic mechanism in suppression of retroelement activity [71]. Second, retrotransposition can be mutationally inactivated via cytosine deamination (C to U transition) operated by members of the APOBEC (apolipoprotein B mRNA-editing enzyme catalytic polypeptide 3, APOBEC3) family [72–74]. APOBEC proteins exert indeed a repressive effect on a broad spectrum of elements during their retrotransposition cycle. Third, another powerful level of control is mediated by RNA interference (RNAi)-related mechanisms that regulate retrotransposon repression, ultimately controlling DNA methylation of retroelements and heterochromatin formation [70]. An interesting mechanism has recently been identified in mice, in which RNAi functions at a post-transcriptional level and ultimately determines LINE-1 mRNA degradation using naturally occurring siRNAs that are processed from bidirectional sense-antisense transcripts [75].

The data on the requirement for retransposon activity in embryogenesis, and the detrimental effects of their uncontrolled activity in differentiated somatic cells, have important implications. They suggest that retrotransposition repressive mechanisms, however diversified at the mechanistic level, have windows in which they are collectively active or inactive: (i). physiologically, they do not operate in early embryogenesis; (ii). they are fully active in normal somatic tissues; and (iii). are lost or deregulated in cancer cells. The body of data summarized thus implicate the loss of these repressive mechanisms as an important step in tumorigenesis, though still much progress remains to be made to fully elucidate the underlying mechanisms at the molecular level.

## 4. LINE-1- and HERV-K-Encoded Reverse Transcriptases Play Differential Roles in Tumor Progression

The implication of LINE-1-encoded RT in tumorigenesis raises the question of whether the RT encoded by HERVs, the other large retrotransposon family, has similar roles. In our laboratory we have addressed that question using a similar RNAi approach to that used for LINE-1 elements: we infected A-375 cells with a retroviral vector expressing HERV-K-specific interfering RNAs to stably down-regulate the expression of HERV-K, which is highly expressed in melanoma [76,77]. In sharp contrast with the effects of LINE-1 interference, down-regulation of HERV-K had no obvious consequence on cell proliferation or differentiation, leaving the interfered cells functionally and morphologically undistinguishable from non-interfered controls [47]. In synthesis, therefore, the data point out that LINE-1-encoded RT: (i). has a central and unique role in human tumorigenesis; (ii). cannot be replaced by RT encoded by other retroelement families; (iii). in particular, is not functionally equivalent to the RT enzyme encoded by HERV-K retroviruses in melanoma.

While HERV-K is not directly involved in melanoma tumor growth, a functional role has emerged in modulating the plasticity of tumor cells [78]. Briefly, we have characterized a cell line established from a human melanoma (TVM-A12 cell line [79]) that undergoes a spontaneous transition from an adherent to a non-adherent growth phenotype. This transition can be regarded as representative of melanoma progression to a more aggressive stage: it is accompanied by an increased proliferation potential, decreased expression of HLA class I molecules as well as Melan-A/MART-1 antigen and increased colony-forming potential in soft agar. These features are suggestive of increased malignancy, increased metastatic potential and ability to evade the immune system. This transition can be experimentally induced by culturing the cells in starving (low-serum) conditions. Interestingly, these changes are strictly dependent on HERV-K expression and related to massive production of HERV-K-related viral-like particles. Indeed, HERV-K-specific RNAi abolished the phenotypic change potential of melanoma cells and inhibited their transition from growth in monolayer to growth in suspension even in low serum conditions [78]. Building up on that evidence, recent work suggests that environmental stimuli can trigger a HERV-K-dependent process through which TVM-A12 melanoma cells undergo differential morphological fates. The differentiating features are again not acquired permanently, and cells revert to their original phenotype when returned to culture in standard medium. This ample cellular plasticity is suggestive of an intrinsic stemness quality in this melanoma cell line; we have recently observed that down-regulation of HERV-K expression in TVM-A12 cells abrogates their stem potential.

It emerges from these data that LINE-1 and HERV families play distinct and complementary roles: LINE-1 has a master function in global control of the developmental program and in establishing the state and fate of cells (*i.e.*, normal differentiated, embryonic undifferentiated or tumorigenic de-differentiated). This control involves genome-wide phenomena and a global resetting of gene expression profiles. HERVs act in more specific pathways responsible for localized cell morphological and functional variations modulatable by environmental signals and stimuli. Consistent with this complementarity of functions, LINE-1 has a characteristic “polarized” expression pattern, with high expression in undifferentiated tissues and low or no activity in differentiated cells ([80] and references herein). In contrast, HERV families show tissue-specific expression: specific families are differentially expressed in different tissue types, yet tend to be expressed in all stages of differentiation in the tissue in which they are active [81–83], with proposed roles in ontogenesis and organ differentiation [84].

## 5. RT Inhibition in Anti-Cancer Differentiation Therapy

The advent of the highly active antiretroviral therapy (HAART) [85], a combination of reverse transcriptase and HIV-protease inhibitors, has had tremendous positive impact not only on treatment of AIDS infection but also on the incidence of AIDS-related tumors. Epidemiological reports show that the incidence of AIDS-related cancers (Kaposi sarcoma, non-Hodgkin lymphoma and invasive cervical cancer) is significantly reduced in HAART-treated patients (see [86,87] and references therein).

As recalled above, our own studies provide evidence, both *in vitro* and *in vivo* in murine models, that non-nucleoside RT inhibitors nevirapine and efavirenz have powerful anti-proliferative and differentiating effects of potential use in a novel cancer therapy. In addition to our own pre-clinical data, recent case reports provide novel proof of concept: indeed, nevirapine treatment of a patient with dedifferentiated metastatic thyroid carcinoma restored thyroglobulin expression and radioiodine uptake and also caused the regression of metastatic lesions [88,89].

The results obtained in AIDS treatment highlight the potential of antiretroviral drugs as attractive and potentially productive tools in the development of new cancer therapeutic drugs. Other HAART components, including nucleoside RT inhibitors and HIV protease inhibitors, have also been tested for cancer therapeutics with variable success [90]. Some of them, particularly protease inhibitors, exert anti-cancer effects independent of the immune reconstitution, confirming the inherent anti-cancer potential of these molecules.

Current cytotoxic therapies to treat cancer have severe secondary effects and non-specific toxicity. Nonnucleoside RT inhibitors may offer a non-toxic or less toxic approach to cancer treatment aimed at inducing cancer cell differentiation and inhibiting metastatic growth. Therapies of this type, termed ‘differentiation therapy’ [91], are based on the assumption that neoplastic cells exhibit altered differentiation features and that treatment with RT inhibitors can trigger their reprogramming, characterized by loss of proliferative capacity and induction of terminal differentiation.

## 6. An RT-Dependent Mechanism in the Genesis and Progression of Cancer

In the continuing search for cancer ethiological causes and effective therapies, cancer has been viewed at various times as a genetic [92–94], genomic [95,96], epigenetic [97,98], evolutionary [99,100] and differentiative [101] disease. These definitions reflect the perspectives under which cancer aspects were being examined. At present, it is difficult to draw a clear-cut conclusion: Cancer emerges as a complex disease in which all these aspects are at work, albeit to a different extent.

The studies summarized here suggest a new model, in which tumorigenesis arises in consequence of the erroneous activation of an RT-dependent mechanism that generates genome-wide chromatin alterations and a global reprogramming of gene expression profiles. In the model, LINE-1 retrotransposons and possibly other retroelement families, including HERV-K, constitute the building blocks of genome-wide regulatory circuits. These circuits would build up during early preimplantation embryonic development and include batteries of coding and non-coding genes. LINE-1 elements are the main source of RT activity in embryos, where their activity is required for early embryo cell division and developmental progression. Physiologically, the endogenous RT is activated at fertilization, continues to operate in early embryogenesis, and is silenced in adult non-pathological tissues. When erroneously reactivated in somatic cells of adult tissues, LINE-1 and the RT activity which they encode become causative agents of tumor onset and progression, promoting de-differentiation and increasing the proliferation potential of cells, paralleled by a global reprogramming of gene expression, in which the “adult” expression profile reverts back to an “embryo-like” profile. This is a crucial aspect of the RT-dependent mechanism, in keeping with the notion that tumor growth reflects the reactivation of embryonic genetic programs.

It is worth stressing that the historical prediction that an embryonic-like transcription “mode” is reactivated in tumor cells [41,42] has recently found ample experimental support. Indeed, numerous embryonic genes are re-expressed in cancer cells; among those, to mention but a few, are OCT4 and other typical preimplantation genes [102], “classical” developmental genes such as Homeobox and Twist family members ([103–106] and references therein), and several genes active in the ontogenesis of organs and tissues [107,108]. Furthermore, studies in a mouse model based on a broad array analysis suggest that transcription in tumors strikingly recapitulates embryonic developmental patterns [109]. As recalled above, the RT-dependent machinery acts at the genome-wide level and introduces non-random structural changes within the nuclear architecture during pathological (tumorigenesis) and physiological (embryogenesis) growth: these changes shape gene expression programs that drive embryo development or tumorigenesis.

The retrotransposon-encoded RT machinery, in our view, integrates two long-standing predictions of regulatory models: first, McClintock's first visionary hypothesis of “controlling elements”, *i.e.*, mobile transposons as key modulators of gene expression [110]; at a later stage, Britten and Davidson suggested that repetitive DNA sequences scattered in the genome form networks exerting regulatory functions on batteries of coding genes [111,112]. Both models evoke an initial genetic level of control (e.g., via insertion of particular elements at specific genomic sites, triggering the ensuing activation of specific gene profiles). Once established, the functional control of these circuits relies entirely on epigenetic mechanisms. Exposure of tumorigenic cells to non-nucleoside RT inhibitors would block this process, restore the “adult-like” gene expression profile and reset the differentiated cell phenotype.

This model is in contrast with the view that cancer is the consequence of highly individualized gene alterations. Rather, it suggests that the genesis of most tumors is caused by the ectopic reactivation of a genome-wide-operating embryonic machinery. The evidence that this machinery is constituted by retroelements points out the increasingly recognized relevant role of the non-coding portion of the genome in the genesis and progression of cancer [113].

## Figures and Tables

**Table 1.** Differentiation of human tumorigenic cell lines by reverse transcriptase (RT) inhibitors.

**Cell line**	**Tumor derivation**	**Differentiation markers**	**Reference**
NB40, HL60	acute promyelocytic leukemia	CD15 myeoloid marker, NBT assay a	[43]
HL60	acute myelocytic leukemia	CD15 myeoloid marker, NBT assay a	[43]
A-375	melanoma	E-cadherin	[45]
PC3	prostate carcinoma	PS-A, androgen receptor	[45]
ARO, FRO	thyroid anaplastic carcinoma	thyroglobulin, thyroglobulin receptor	[46]

a Nitroblue tetrazolium (NBT) dye reduction assay.

**Table 2.** Activation of retrotransposons in cancer.

**Tumor Type**	**Reference**
Testicular tumor	[51]
Urothelial bladder carcinoma	[52,53]
Prostate carcinoma	[53]
Hepatocellular carcinoma	[54]
Chronic lymphocytic leukemia	[55]
Chronic myeloid leukemia	[56]
